# HbA1C Variability Is Strongly Associated with Development of Macroalbuminuria in Normal or Microalbuminuria in Patients with Type 2 Diabetes Mellitus: A Six-Year Follow-Up Study

**DOI:** 10.1155/2020/7462158

**Published:** 2020-01-24

**Authors:** Wen-Chan Chiu, Yun-Ru Lai, Ben-Chung Cheng, Chih-Cheng Huang, Jung-Fu Chen, Cheng-Hsien Lu

**Affiliations:** ^1^Departments of Rheumatology, Allergy, and Immunology, Internal Medicine Kaohsiung Chang Gung Memorial Hospital, Chang Gung University College of Medicine, Kaohsiung, Taiwan; ^2^Department of Biological Science, National Sun Yat-Sen University, Kaohsiung, Taiwan; ^3^Department of Neurology, Kaohsiung Chang Gung Memorial Hospital, Chang Gung University College of Medicine, Kaohsiung, Taiwan; ^4^Division of Nephrology, Internal Medicine Kaohsiung Chang Gung Memorial Hospital, Chang Gung University College of Medicine, Kaohsiung, Taiwan; ^5^Division of Metabolism, Internal Medicine Kaohsiung Chang Gung Memorial Hospital, Chang Gung University College of Medicine, Kaohsiung, Taiwan; ^6^Center for Shockwave Medicine and Tissue Engineering, Kaohsiung Chang Gung Memorial Hospital, Chang Gung University College of Medicine, Kaohsiung, Taiwan; ^7^Department of Neurology, Xiamen Chang Gung Memorial Hospital, Xiamen, Fujian, China

## Abstract

**Background:**

Glycemic variability is associated with higher risk of microvascular complications in patients with type 2 diabetes.

**Aim:**

To test the hypothesis that glycemic variability can contribute to progression to macroalbuminuria in normal or microalbuminuria in patients with type 2 diabetes.

**Design:**

This prospective study enrolled 193 patients with type 2 diabetes at a tertiary medical center.

**Methods:**

For each patient, the intrapersonal glycemic variability (mean, SD, and coefficient of variation of HbA1c) was calculated using all measurements obtained three years before the study. Patients were divided into four groups stratified by both urine albumin/creatinine ratio and HbA1c-SD. The presence of macroalbuminuria was assessed with Kaplan–Meier plots and compared by log-rank test.

**Results:**

Of the 193 patients, 83 patients were in the macroalbuminuria state. Patients in the initial macroalbuminuria group after enrollment had the highest diabetes duration, mean, CV-HbA1c and HbA1c-SD, and uric acid level, and the lowest estimate glomerular filtration rate, followed by subsequent macroalbuminuria and without macroalbuminuria groups. Patients with microalbuminuria and high HbA1c-SD showed the highest progression rate to macroalbuminuria, after a six-year follow-up study by Kaplan–Meier Plots and compared by log-rank test.

**Conclusions:**

Higher HbA1C variability is more likely to progress to macroalbuminuria in those patients who are already in a microalbuminuria state. We recommend that clinicians should aggressively control blood glucose to an acceptable range and avoid blood glucose fluctuations by individualized treatment to prevent renal status progression.

## 1. Background

Aggressive control of blood glucose is pivotal for patients with type 2 diabetes, which can prevent microvascular and macrovascular complications [1, 2]. There is compelling evidence that the level of long-term hyperglycemia influences the risk of microvascular disease in patients with type 1 and 2 diabetes [1, 2]. Glycemic variability (GV) is a term used to describe impairment of glycemic control. Short-term GV indicated that patients with similar mean glucose or HbA1c values can show markedly different daily glucose profiles. Long-term GV assessment using HbA1c variability is also an independent risk factor for the development of microvascular complications in those with type 1 and 2 diabetes [3, 4].

The UK Prospective Diabetes Study investigated the development and progression of the stages of diabetic kidney diseases (DKD) (microalbuminuria, macroalbuminuria, persistently elevated plasma creatinine or renal replacement therapy, and death) and found that individuals with macroalbuminuria were more likely to die in any year than to develop renal failure [5]. To date, there is a paucity of information that focuses on estimating the relationship between long-term GV (HbA1c variability) and the development of overt nephropathy (macroalbuminuria) in patients with type 2 diabetes. The clinical study showed that multifactorial treatment can slow the progression to nephropathy and renal function loss, reducing the risk of end-stage renal disease [6]. Therefore, exploring the role of HbA1c variability on the subsequent severity of overt nephropathy can lead to development of therapeutic strategies, which are beneficial to patients with diabetes. Therefore, potential risk factors need to be delineated to determine patients who are most appropriate for aggressive treatment. In this study, we tested the hypothesis that HbA1c variability is a prognostic factor that can influence the development of overt nephropathy (macroalbuminuria) in patients with type 2 diabetes after a six-year follow-up study. The successful translation of these approaches offers the promise of reducing overt nephropathy (macroalbuminuria) and improving the quality of life in patients with type 2 diabetes.

## 2. Patients and Methods

### 2.1. Patients

In total, 238 patients (age range, 29–90 years) with type 2 diabetes who visited the outpatient diabetic clinic at Kaohsiung Chang Gung Memorial Hospital in Taiwan were identified. Exclusion criteria included the following: (1) less than four HbA1c measurements and (2) end-stage renal diseases (chronic kidney disease Stage 4 or 5). Thus, only 193 participants were enrolled in the study. The study was approved by the Ethics Committee of Chang Gung Memorial Hospital Institutional Review Board (201701243B0 and 201800388B0).

### 2.2. Baseline Clinical and Laboratory Measurements

All patients underwent complete physical examinations upon enrollment and at their subsequent follow-up at the outpatient clinic. A detailed medical history regarding prior use of medications was obtained from patients through standardized questions. Baseline demographic data of patients and underlying disease (coronary artery disease (CAD), ischemic stroke, and diabetic retinopathy (DR)), and laboratory parameters were obtained at baseline [7].

### 2.3. Assessment of Albuminuria and Glomerular Filtration Rate

The normal rate of albumin excretion is less than 30 mg/day; therefore, persistent albumin excretion between 30 and 300 mg/day is called moderately increased albuminuria (microalbuminuria) [8] and albumin excretion above 300 mg/day is considered severely increased albuminuria (macroalbuminuria [8], which is also called overt proteinuria) [9]. The abbreviated Modification of Diet in Renal Disease study formula recalibrated for patients was used to estimate glomerular filtration rate (eGFR): eGFR = 186 × [serum creatinine (mmol/L) × 0.011]^−1.154^ × (age)^−0.203^ × (0.742 for women) × 1.233, where 1.233 is the adjusting coefficient for Chinese patients [8].

### 2.4. Assessment of Glycemic Variability

The intrapersonal mean, SD, and coefficient of variation [CV = HbA1c-SD/(0.1 × mean HbA1c)] of HbA1c were calculated using all measurements obtained three years prior to the study (baseline) for each patient. The level of HbA1c was evaluated regularly at an outpatient clinic every three to six months. HbA1c-SD was considered a measure of glycemic variability and coefficient of variation (CV) as a normalized measure of glycemic variability. Because the number of individual visits could influence the HbA1c-SD (with fewer visits likely to artificially inflate SD), values for HbA1c-SD were divided by [*n*/(*n* − 1)]^0.5^, where *n* is the number of HbA1c measurements, to minimize any effect of different numbers of HbA1c measurements on the calculated values [7, 10].

### 2.5. Outcome Measurement

In this study, mean HbA1c and glycemic variability (HbA1c-SD and CV HbA1c) were calculated using all measurements obtained three years before the study (baseline). Since both underlying DKD stage and GV could be the risks factors that contribute to DKD progression, patients were divided into four groups according to urine albumin/creatinine ratio (UACR) and HbA1c-SD (i.e., UACR <30 mg/gm, UACR between 30 and 300 mg/gm, <median HbA1c-SD, and ≥median HbA1c-SD). To avoid interference with statistical analysis, the 83 patients who already had macroalbuminuria were excluded from evaluation of risk factors associated with macroalbuminuria progression. Finally, there were 110 participants enrolled in this study. The endpoint of the survival curve is defined as a urine Albumin/Creatinine Ratio (UACR) above 300 after a six-year follow-up.

### 2.6. Statistical Analysis

Data are expressed as means ± standard derivations (SDs) or medians (interquartile ranges). Categorical variables were compared using Chi-square or Fisher's exact tests. Continuous variables that were not normally distributed were logarithmically transformed to improve normality and comparisons. Three steps of statistical analyses were performed. First, trends across more than two groups were analyzed using linear polynomial contrasts (ANOVA) for normally distributed variables. Second, risk factors for subsequent macroalbuminuria that occurred after the six-year follow-up period were determined. Third, the association among the four groups (normal albuminuria with low HbA1c-SD, normal albuminuria with high HbA1c-SD, microalbuminuria and low HbA1c-SD, and microalbuminuria and high HbA1c-SD) and the survival curve between presence of macroalbuminuria were assessed with Kaplan–Meier plots and compared by log-rank test. All statistical analyses were conducted using the SAS software package, version 9.1 (2002, SAS Statistical Institute, Cary, North Carolina).

## 3. Results

### 3.1. Baseline Characteristics in Patients with Diabetes

**Figure 1:**
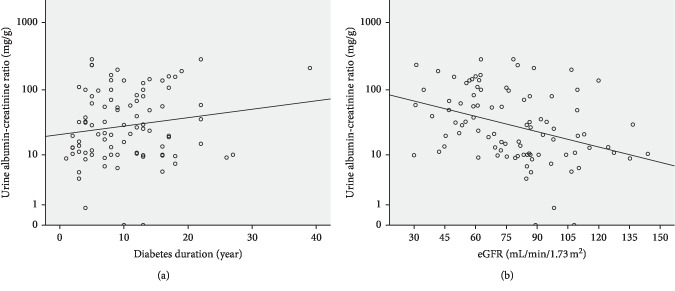
Relationship between urine albumin-creatinine ratio and (a) diabetes duration and (b) eGFR in patients with type 2 diabetes.

Of the 193 patients with diabetes, 66 were women (age range, 37–79 years; mean age = 64.2 years) and 127 were men (age range, 29–90 years; mean age = 62.6 years). The baseline characteristics, underlying diseases, types of diabetes medications, and cardio-metabolic parameters are presented in Supplementary Table, stratified by initial, subsequent, and without macroalbuminuria after a six-year follow-up study. Among them, there were 83 patients with macroalbuminuria. Patients in the initial macroalbuminuria group after enrollment had the highest diabetes duration (*P*=0.007), mean, CV-HbA1c, HbA1c-SD (*P*=0.002, *P*=0.001, and *P* < 0.001, respectively), and uric acid levels (*P* < 0.001), followed by the subsequent macroalbuminuria and without macroalbuminuria groups. Patients in the initial macroalbuminuria group after enrollment had lower eGFR (*P* < 0.001), followed by the subsequent macroalbuminuria and without macroalbuminuria groups. UACR was positively correlated with diabetes duration (Figure 1(a)) but negatively correlated with eGFR (Figure 1(b)) in patients with type 2 diabetes.

### 3.2. Characteristics in Patients without Initial Macroalbuminuria Stratified by Both Urine Albumin/Creatinine Ratio and Glycemic Variability

**Figure 2:**
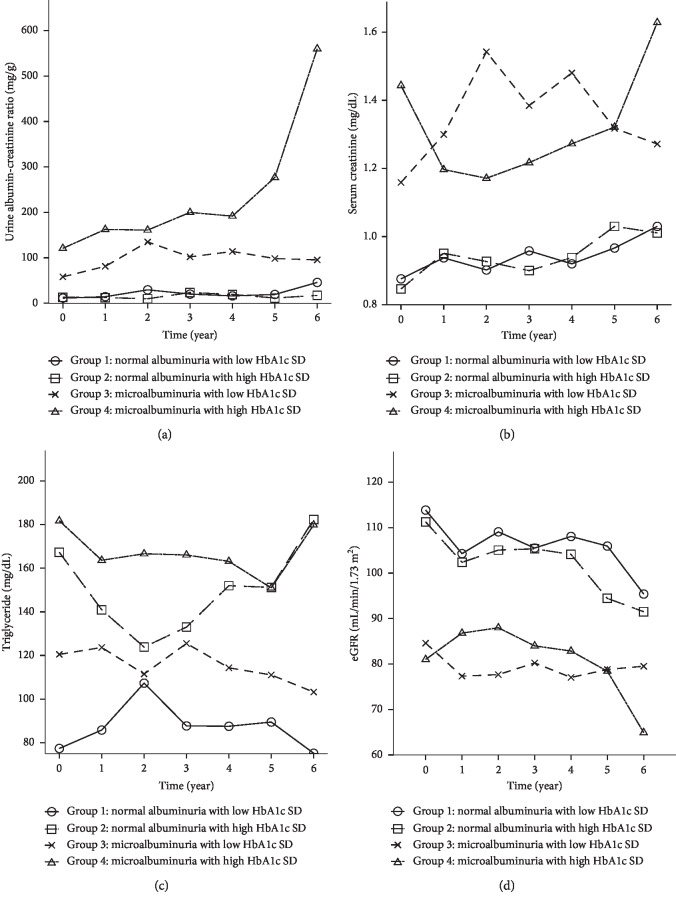
The level of urine albumin-creatinine ratio (a), serum creatinine (b), triglyceride (c), and eGFR (d) among the four groups during the follow-up period.

We excluded the 83 patients who already had macroalbuminuria and divided the remaining 110 patients stratified by both UACR and glycemic variability into four groups (normal albuminuria with low HbA1c-SD (Group 1), normal albuminuria with high HbA1c-SD (Group 2), microalbuminuria and low HbA1c-SD (Group 3), and microalbuminuria and high HbA1c-SD (Group 4)) (Tables 1 and 2). The cut-off value of median HbA1c-SD in those patients with normal albuminuria (UACR <30 mg/gm) with low/high HbA1c-SD and microalbuminuria with low/high HbA1c-SD (UACR between 30 and 300 mg/gm) is 0.32 and 0.92, respectively. Patients with both microalbuminuria and high HbA1c-SD had the highest mean, CV HbA1c, and triglyceride levels. Conversely, patients with both normal albuminuria with low HbA1c-SD had the lowest mean, CV HbA1c, and triglyceride levels (*P* < 0.001, *P* < 0.001, and *P*=0.002, respectively). The level of UACR (Figure 2(a)), serum creatinine (Figure 2(b)), triglyceride (Figure 2(c)), and eGFR (Figure 2(d)) among the four groups during the follow-up period were listed in Figure 2. Those patients with microalbuminuria and high HbA1c-SD (Group 4) are most prone to increased UACR and serum creatinine level and also decreased eGFR level during the six-year follow-up period.

### 3.3. Risk Factors of Progression to Macroalbuminuria in Patients with Type 2 Diabetes

**Figure 3:**
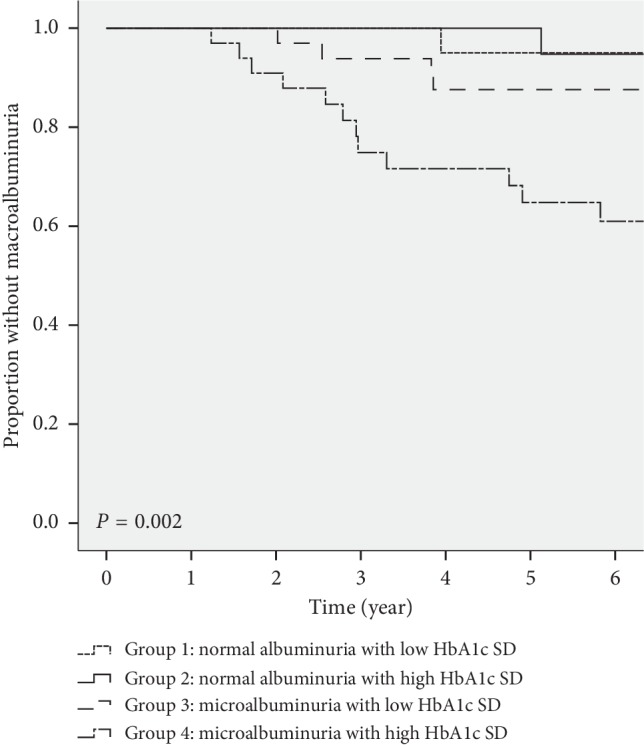
Kaplan–Meier plots indicating the percentage of macroalbuminuria in 110 patients with type 2 diabetes mellitus without initial macroalbuminuria. The patients were divided into four groups (normal albuminuria with low HbA1c-SD, normal albuminuria with high HbA1c-SD, microalbuminuria and low HbA1c-SD, and microalbuminuria and high HbA1c-SD). The *P* value was obtained by log-rank comparison of data (Log-rank *P* value = 0.002).

The percentages of macroalbuminuria development in the four groups (normal albuminuria with low HbA1c-SD, normal albuminuria with high HbA1c-SD, microalbuminuria and low HbA1c-SD, and microalbuminuria and high HbA1c-SD) were 5%, 5%, 14.3%, and 34.3%, respectively. The death percentages in the four groups (normal albuminuria with low HbA1c-SD, normal albuminuria with high HbA1c-SD, microalbuminuria and low HbA1c-SD, and microalbuminuria and high HbA1c-SD) were 5%, 5%, 8.6%, and 5.7%, respectively (Table 1). To examine both urine UACR and glycemic variability, we divided the 110 patients without initial macroalbuminuria into four groups (normal albuminuria with low HbA1c-SD, normal albuminuria with high HbA1c-SD, microalbuminuria and low HbA1c-SD, and microalbuminuria and high HbA1c-SD). Subsequently, we calculated Kaplan–Meier estimates of the fraction developing macroalbuminuria at different times for each subgroup and used a log-rank test to measure statistical significance among the four groups. The statistical results showed that patients who had microalbuminuria and high HbA1c-SD had the highest progression rate to macroalbuminuria, followed by microalbuminuria and low HbA1c-SD, normal albuminuria with low HbA1c-SD, and normal albuminuria with high HbA1c-SD after a six-year follow-up study (Log-rank *P* value 0.002) (Figure 3).

## 4. Discussion

### 4.1. Major Findings of Our Study

Our study confirmed the hypothesis that HbA1C variability is strongly associated with the development of macroalbuminuria, especially patients under a microalbuminuria state. We examined the role of HbA1C variability in normal or microalbuminuria patients with type 2 diabetes after a six-year follow-up and discovered four major findings. First, patients with initial macroalbuminuria after enrollment had the highest diabetes duration, mean, CV-HbA1c, HbA1c-SD, and uric acid levels (*P* < 0.001), followed by the subsequent macroalbuminuria and without macroalbuminuria groups. Second, patients in the initial macroalbuminuria group after enrollment had lower eGFR, followed by the subsequent macroalbuminuria and without macroalbuminuria groups. Third, patients with both microalbuminuria and high HbA1c-SD had the highest mean, CV HbA1c, and triglyceride levels. Conversely, patients in the normal albuminuria with low HbA1c-SD group had the lowest mean, CV HbA1c, and triglyceride levels. Fourth, higher HbA1C variability is more likely to progress to macroalbuminuria especially in those patients who are already in a microalbuminuria state.

### 4.2. Relationship between HbA1C Variability and Renal Status Progression

Fluctuating or persisting high glucose levels can induce oxidative stress, overproduction of reactive oxygen species, and endothelial dysfunction and contribute to microvascular (nephropathy, retinopathy, and neuropathy) in patients with type 2 diabetes [11, 12]. Our recent studies also demonstrated that and HbA1c variability is also considered a prognostic factor, in both diabetic peripheral neuropathy and cardiovascular autonomic neuropathy [7, 13].

The United Kingdom Prospective Diabetes Study demonstrated that progression to microalbuminuria occurred at the rate of 2.0% annually and from microalbuminuria to macroalbuminuria at the rate of 2.8% annually from the diagnosis of diabetes [5]. This study also demonstrated that relatively fewer patients developed macroalbuminuria. However, the death rate exceeded the rate of progression to worse nephropathy in subsequent macroalbuminuria patients. Further, our study showed that patients in the initial macroalbuminuria group after enrollment had the highest diabetes duration, followed by the subsequent macroalbuminuria and without macroalbuminuria groups. Higher HbA1C variability is strongly associated with higher microalbuminuria progression to macroalbuminuria. Besides the natural course of diabetes duration, the increased pathology load could also be a result of treatment paradigms. Therefore, aggressive blood glucose control to an acceptable range and avoidance of blood glucose fluctuations through individualized treatment can prevent further nerve damage [11, 12].

Additionally, studies on risk factors and the role of HbA1C variability in microalbuminuria development and renal status progression in diabetes are needed. One meta-analysis study investigated the relationship between HbA1C variability and the risk of renal status progression in diabetes mellitus [13]. The analysis showed that HbA1c variability was independently associated with the development of microalbuminuria and progression of renal status in both type 1 and 2 diabetes patients, which include five type 2 diabetic and four type 1 diabetic patients enrolled in the study [4, 13–16]. The mean follow-up durations were between 4.3 and 7.2 years. HbA1c variability was expressed by CV HbA1c in one, HBA1C-SD in seven, and both in one study [4, 13–17]. Two clinical studies examined the association between HbA1c variability and microalbuminuria development [4, 17]. Further, two studies determined the influence of HbA1c variability on the risk of nephropathy progression [14, 16]. Another study evaluated HbA1c variability with incident chronic kidney disease (estimated glomerular filtration rate <60 mL/min per 1.73 m^2^) and cardiovascular disease (events of ischemic heart disease, heart failure, ischemic stroke, or peripheral vascular disease) [15]. Our study evaluated the role of HbA1c variability on macroalbuminuria progression in normal or microalbuminuria patients with type 2 diabetes. The discrepancy between these studies and our study may be attributed to different diabetes duration, renal outcomes (e.g., microalbuminuria, eGFR <60, presence of nephropathy, and presence of macroalbuminuria), HbA1c variability (e.g. CV HbA1c or HbA1c-SD), and statistical methods.

The role of glycemic variability for DKD may provide important clues to etiologies, or merely reflect chance associations [3, 4, 18, 19]. One study measured the mean amplitude of glycemic excursions (MAGE) and HbA1c SD over a 2-year period, as the indicators of short and long-term glycemic variability, respectively [19]. This study found both short- and long-term glycemic variability can increase oxidative stress and chronic inflammation [19]. Evidence from post hoc analysis of the Diabetes Control and Complications Trial of subjects with type 1 diabetes also show an association between HbA1c variability and the microvascular complications of diabetes, suggesting that longer-term fluctuations in glycemia seem to contribute to the development of retinopathy and nephropathy in type 1 diabetes [20]. Our study also demonstrates that higher HbA1C variability is more likely to progress to macroalbuminuria in those type 2 diabetes patients who are already in a microalbuminuria state after a six-year follow-up study. Although the impact of HbA1c variability on oxidative stress and reactive oxygen species generation has not been studied from our study, the excessive HbA1c SD seems to induce oxidative stress and chronic inflammation, and it finally contributes to the progression of macroalbuminuria. Future study is expected to clarify the pathway.

### 4.3. Study Limitations

This study has several limitations. First, we excluded patients who had initial macroalbuminuria and HbA1c measurements of less than four. Thus, there is uncertainty in assessing the role of HbA1c variability in unselected type 2 diabetes. Second, the follow-up duration in our study is not long and sample sizes are not large; therefore, we cannot clarify other confounding factors (e.g., diabetes duration) on the development of microalbuminuria and/or macroalbuminuria and the progression of renal status.

## 5. Conclusion

Higher HbA1C variability is more likely to progress to macroalbuminuria in those patients who are already in a microalbuminuria state. As HbA1c variability is considered a prognostic factor, aggressive control of blood glucose to an acceptable range and avoidance of blood glucose fluctuations through more individualized treatment can prevent further development of macroalbuminuria and the progression of renal status in diabetes.

## Figures and Tables

**Table 1:** Characteristics of patients without initial macroalbuminuria stratified by both urine albumin/creatinine ratio and HbA1c variability.

	UACR< 30 mg/gm		UACR between 30 to 300 mg/gm		*P* value
	<median HbA1c-SD^Ϯ^ (*n* = 20) group 1	≥median HbA1c-SD^Ϯ^ (*n* = 20) group 2	<median HbA1c-SD^Ϯ^ (*n* = 35) group 3	≥median HbA1c-SD^Ϯ^ (*n* = 35) group 4	
*Characteristics*					
Age (year)	61.2 ± 9.99	59.8 ± 5.75	65.5 ± 7.12	60.9 ± 9.81	0.05
Sex (female/male)	4/16	8/12	10/25	10/25	0.59
Diabetes duration (year)	8.7 ± 7.17	10.8 ± 6.06	11.5 ± 7.57	10.4 ± 6.04	0.51
Height (cm)	163.6 ± 7.32	162.3 ± 8.18	162.9 ± 8.16	163.7 ± 8.22	0.92
Body weight (Kg)	66.8 ± 10.79	69.5 ± 12.49	68.7 ± 11.58	71.4 ± 10.93	0.52
Body mass index	25.0 ± 2.81	26.3 ± 3.98	25.6 ± 3.11	26.4 ± 3.45	0.42
Waist circumstance (cm)	87.4 ± 11.28	94.3 ± 7.74	94.3 ± 11.15	94.1 ± 7.76	0.12
SBP (mmHg)	136 ± 20.42	139.3 ± 17.75	132.5 ± 14.64	137.8 ± 17.31	0.47
DBP (mmHg)	75.1 ± 8.81	76.7 ± 11.63	70.5 ± 9.7	76.5 ± 9.43	0.05

*Baseline underlying disease*					
Hypertension	12 (60)	16 (80)	30 (85.7)	26 (74.3)	0.18
Coronary heart disease	3 (15)	4 (20)	5 (14.3)	4 (11.4)	0.86
Ischemic stroke	2 (10)	2 (10)	4 (11.4)	4 (11.4)	1.0
Retinopathy	1 (5.3)	4 (20)	11 (32.4)	12 (37.5)	0.06
Six-year follow-up outcome					
Percentage of macroalbuminuria, *n* (%)	1 (5)	1 (5)	5 (14.3)	12 (34.3)	**0.009**
Death	1(5)	1 (5)	3 (8.6)	2 (5.7)	0.933

Data are presented as means ± standard deviations or *n* (%). Abbreviations: *n*, number of cases; UACR, urine albumin/creatinine ratio; SBP, systolic blood pressure; DBP, diastolic blood pressure; OHA, oral hypoglycemic agent; ACE, angiotensin-converting enzyme; ARB, angiotensin II receptor blocker; OHA. Ϯ = the value of median HbA1c-SD in the two groups (UACR <30 mg and UACR between 30–300 mg) was 0.32 and 0.92, respectively.

**Table 2:** Laboratory test findings of patients without initial macroalbuminuria stratified by both urine albumin/creatinine ratio and HbA1c variability.

	UACR <30 mg/gm		UACR between 30 to 300 mg/gm		*P* value
	<median HbA1c-SD^Ϯ^ (*n* = 20) Group 1	≥median HbA1c-SD^Ϯ^ (*n* = 20) Group 2	<median HbA1c-SD^Ϯ^ (*n* = 35) Group 3	≥median HbA1c-SD^Ϯ^ (*n* = 35) Group 4	
Total cholesterol (mmol/L)	155.5 ± 25.2	150.6 ± 26.2	153.6 ± 26.4	167.1 ± 37.2	0.16
Triglyceride (mmol/L)	84.3 ± 32.4	153.5 ± 85.3	112.1 ± 73.5	156.5 ± 87.0	**0.002**
HDL-C (mmol/L)	57.2 ± 11.16	49.5 ± 11.53	56.6 ± 14.09	52.4 ± 18.4	0.24
LDL-C (mmol/L)	81.5 ± 20.3	71.5 ± 22.2	75 ± 27.8	84.4 ± 34.2	0.32
UA (mmol/L)	6.3 ± 1.4	6.2 ± 1.4	7.1 ± 1.9	6.5 ± 2.3	0.28
hs-CRP (mmol/L)	0.8 ± 0.6	1.3 ± 1.1	1.8 ± 1.3	3.2 ± 2.3	0.22
Mean HbA1c (%)	6.8 ± 0.7	7.4 ± 1.0	7.3 ± 0.9	8.1 ± 0.9	**<0.001**
CV HbA1c (%)	5.7 ± 1.48	12.9 ± 5.96	8.1 ± 2.6	16.9 ± 4.7	**<0.001**
HbA1c-SD (%)	0.4 ± 0.09	1 ± 0.43	0.6 ± 0.2	1.3 ± 0.4	**<0.001**
Urine albumin/creatinine ratio (mg/mg)	0 ± 0.01	0 ± 0.01	0.1 ± 0.08	0.1 ± 0.06	**<0.001**
eGFR (mL/min/1.73 m^2^)	92 ± 25.2	88.3 ± 16.8	76.9 ± 22.2	68.4 ± 25.8	**0.001**

Data are presented as means ± standard deviations or *n* (%). Abbreviations: *n*, number of cases; UACR, urine albumin/creatinine ratio; SBP, systolic blood pressure; DBP, diastolic blood pressure; OHA, oral hypoglycemic agent; ACE, angiotensin-converting enzyme; ARB, angiotensin II receptor blocker; HDL-C, high-density lipoprotein cholesterol; LDL-C, low-density lipoprotein cholesterol; UA, uric acid; hsCRP, high-sensitive C-reactive protein; HbA1c, glycohemoglobin; eGFR, estimated glomerular filtration rate; CV, coefficient of variation; Ϯ = The value of median HbA1c-SD in the two groups (UACR <30 mg and UACR between 30–300 mg) was 0.32 and 0.92, respectively.

## Data Availability

The data used to support the findings of this study are available from the corresponding author upon request.

## Abbreviations

CV: Coefficient of variation
DKD: Diabetic kidney diseases
GV: Glycemic variability
CAD: Coronary artery disease
DR: Diabetic retinopathy
eGFR: Estimate glomerular filtration rate (eGFR)
SD: Standard derivations
UACR: Urinary albumin-to-creatinine ratio.

## Supplementary Materials

Supplementary Materials Supplementary Table: characteristics of patients with initial, subsequent, or without macroalbuminuria (urine albumin/creatinine ratio above 300 mg) during six-year follow-up study.
