# Supplementary material for: HbA1C Variability Is Strongly Associated with Development of Macroalbuminuria in Normal or Microalbuminuria in Patients with Type 2 Diabetes Mellitus: A Six-Year Follow-Up Study
Source: Biomed Res Int. 2020 Jan 24;2020:7462158. doi: 10.1155/2020/7462158 (PMC7003285; doi:10.1155/2020/7462158)
Supplement: Supplementary Materials — Supplementary Table: characteristics of patients with initial, subsequent, or without macroalbuminuria (urine albumin/creatinine ratio above 300 mg) during six-year follow-up study. [file 7462158.f1.doc]

***Supplementary Table. Characteristics of patients with initial, subsequent, or without macroalbuminuria (Urine Albumin/Creatinine Ratio above 300 mg) during six-year follow-up study***

|  | *Initial macroalbuminuria*  *(n=83)* | *Subsequent macroalbuminuria (n=19)* | *Without*  *macroalbuminuria*  *(n=91)* | *P-value* |
| --- | --- | --- | --- | --- |
| *Characteristics* |  |  |  |  |
| *Age (year)* | *64.5±9.43* | *63.5±10.37* | *61.9±8.25* | *0.173* |
| *Sex (female/male)* | *34/49* | *4/15* | *28/63* | *0.163* |
| *Diabetes duration (year)* | *13.3±6.87* | *12.5±5.18* | *10.1±6.99* | ***0.007*** |
| *Body mass index* | *26.8±4.17* | *25.1±2.79* | *26±3.44* | *0.159* |
| *Waist circumstance (cm)* | *93.8±11.47* | *92.9±7.52* | *92.9±10.27* | *0.884* |
| *SBP (mmHg)* | *143±19.71* | *136.1±20.61* | *136.1±16.5* | *0.038* |
| *DBP(mmHg)* | *74.8±11.18* | *72.8±11.31* | *74.7±9.84* | *0.76* |
| ***Baseline underlying disease*** |  |  |  |  |
| *Hypertension (%)* | *83(100)* | *16(84.2)* | *68(74.7)* | ***<.001*** |
| *Coronary heart disease (%)* | *15(18.1)* | *5(26.3)* | *11(12.1)* | *0.247* |
| *Ischemic stroke (%)* | *19(22.9)* | *3(15.8)* | *9(9.9)* | *0.066* |
| *Retinopathy (%)* | *35(42.7)* | *6(31.6)* | *22(25.6)* | *0.063* |
| ***Type of diabetes treatment*** |  |  |  |  |
| *Insulin + OHA* | *36(43.4)* | *6(31.6)* | *13(14.3)* | *<.001* |
| *OHA only* | *43(51.8)* | *13(68.4)* | *76(83.5)* | *<.001* |
| *Insulin only* | *3(3.6)* | *0(0)* | *1(1.1)* | *0.407* |
| *No treatment* | *1(1.2)* | *0(0)* | *1(1.1)* | *0.893* |
| ***Other concomitant medications*** |  |  |  |  |
| *ACE inhibitor or ARB* | *71(85.5)* | *16(84.2)* | *59(64.8)* | *0.004* |
| *Beta-blocker* | *35(42.2)* | *7(36.8)* | *22(24.2)* | *0.039* |
| *Calcium channel blocker* | *43(51.8)* | *6(31.6)* | *29(31.9)* | *0.02* |
| *Diuretics* | *59(71.1)* | *11(57.9)* | *37(40.7)* | *<.001* |
| *Antiplatelet medications* | *59(71.1)* | *15(78.9)* | *71(78)* | *0.527* |
| *Lipid-lowering medications* | *78(94)* | *12(63.2)* | *62(68.1)* | *<.001* |
| ***Laboratory test findings*** |  |  |  |  |
| *Total cholesterol(mmol/L)* | *148.8±26.7* | *155.4±28.4* | *158.2±30.9* | *0.103* |
| *Triglyceride(mmol/L)* | *146.4±82.1* | *148±86.3* | *124.7±77.8* | *0.167* |
| *HDL-C (mmol/L)* | *50.1±13.4* | *54±12.6* | *54.1±15.3* | *0.166* |
| *LDL-C (mmol/L)* | *69.7±21.5* | *71.9±26.4* | *79.9±28.4* | ***0.03*** |
| *UA (mmol/L)* | *7.9±1.7* | *7±2.5* | *6.5±1.79* | ***<0.001*** |
| *Mean HbA1c (%)* | *7.9±1* | *7.5±0.85* | *7.3±0.97* | ***0.002*** |
| *CV HbA1c* | *11.3±5.95* | *9.5±4.09* | *8.2±5.47* | ***0.001*** |
| *HbA1c SD* | *0.8±0.44* | *0.7±0.33* | *0.6±0.41* | ***<0.001*** |
| *Urine Albumin-creatinine ratio (mg/mg)* | *1±1.56* | *0.1±0.08* | *0±0.06* | ***<0.001*** |
| *eGFR (mL/min/1.73 m^2^)* | *47.6±23.* | *74.6±26.1* | *79.9±24.3* | ***<0.001*** |
| *Data are presented as means ± standard deviations or n (%).*  *Abbreviations: n, number of cases; SBP, systolic blood pressure; DBP, diastolic blood pressure; OHA, oral hypoglycemic agent; ACE, angiotensin-converting enzyme; ARB, angiotensin II receptor blocker; HDL-C, high-density lipoprotein cholesterol; LDL-C, low-density lipoprotein cholesterol; UA, uric acid; hs-CRP, high-sensitive C-reactive protein; HbA1c, glycohemoglobin; eGFR, estimated glomerular filtration rate; CV, coefficient of variation* | | | | |
